# Evaluation of antibacterial properties of nisin peptide expressed in carrots

**DOI:** 10.1038/s41598-023-49466-7

**Published:** 2023-12-13

**Authors:** Masoumeh Fallah Ziarani, Masoud Tohidfar, Mohammad Hossein Mirjalili

**Affiliations:** 1 Department of Cell & Molecular Biology, Faculty of Life Sciences & Biotechnology, Shahid Beheshti University, Tehran, 1983969411 Iran; 2 Department of Agriculture, Medicinal Plants and Drugs Research Institute, Shahid Beheshti University, Tehran, 1983969411 Iran

**Keywords:** Biotechnology, Microbiology, Plant sciences

## Abstract

Nisin, derived from *Lactococcus lactis*, is a well-known natural food preservative. In the present study, the gene of nisin was transformed to carrot by *Agrobacterium tumefaciens* strain LBA4404 harboring the recombinant binary vector pBI121 containing neomycin phosphotransferase II (*npt*II) gene, peptide signal KDEL, and Kozak sequence. The integration of nisin and *npt*II transgenes into the plant genome was confirmed by polymerase chain reaction (PCR) and dot blot analysis. The gene expression was also performed by RT-PCR and Enzyme-Linked Immunosorbent Assay. The level of nisin expressed in one gram of transgenic plant ranged from 0.05 to 0.08 μg/ml. The stability of nisin varied in orange and peach juices depending on the temperature on the 70th day. The leaf protein extracted from the transgenic plant showed a significant preservative effect of nisin in peach and orange juice. A complete inhibition activity against *Staphylococcus aureus* and *Escherichia coli* in orange juice was observed within 24 h. After 24 h, log 1 and log 2 were obtained in a peach juice containing *Staphylococcus aureus* and *Escherichia coli*, respectively. Results of HPLC indicated that Chlorogenic and Chicoric acid compounds were increased in transgenic plants, but this increase was not significant. The study of determining the genetic stability of transgenic plants in comparison with non-transgenic plants showed high genetic stability between non-transgenic plants and transgenic plants. This study confirmed the significant inhibitory effect of nisin protein on gram-positive and gram-negative bacteria.

## Introduction

The growth of microorganisms generally causes chemical and enzymatic reactions that lead to changes in the color, taste, texture, nutritional value and health of food products. Microbial contamination is a significant cause of food spoilage. Unwanted microbial growth in food is detrimental to human health and the national economy^1^. Therefore, preventing or delaying the various changes in a food product is critically needed. Preservatives are synthetic or natural substances added to food or medicinal products to prevent or reduce the growth of microbes or spoilage during storage^1,2^.

Benzoates, nitrites, sulfites, and sorbates are some examples of chemical preservatives used in food industries^3^. Some food preservatives such as sulfur dioxide and nitrites are harmful. Sulfur dioxide irritates the airways and nitrite is carcinogenic^2^. There are concerns about the side effects of chemical additives. One of the problems with using chemical preservatives is damage to the liver due to the pressure on the liver to expel these preservatives and increase the risk of cancer^2^. In order to solve these problems, natural preservatives have received significant attention today. Natural preservatives are of plant or animal origin or are derived from microbial sources known as green preservatives^4^. Among the natural preservatives, the preservatives produced by microbes have received more attention. Examples of microbial preservatives include acids, bacteriocins, alcohols, and diacetyl^4^.

Many lactic acid bacteria and other gram-positive bacteria produce peptides that have antimicrobial activity^5^. These peptides have a wide range of activities. One class of these antimicrobial peptides that have attracted much attention in recent years is lantibiotics^6^. One of the best lantibiotics currently in use is nisin, produced by several strains of *Lactococcus lactis*^7^*.* Nisin has been widely used as a food preservative for a long time in different countries, and today, due to the tendency of consumers to use biological preservatives, the consumption of these substances in the food industry has increased^7^.

The effect of medicinal plant extracts against gram-positive and gram-negative bacteria has been extensively reported^8–10^. The introduction of genes encoding small antimicrobial peptides when introduced into plants did enhance their resistance to bacterial and fungal pathogens^11–14^. The study by Jagannath et al.^8^ used nisin to stabilize novel fruit and vegetable functional juices. In the research of Qian et al.^15^, the antibacterial effect of nisin protein was confirmed. It was also found that the nisin protein's effect on gram-positive bacteria is more than on gram-negative bacteria^15,16^. Based on the findings of Zheng-Rong et al.^17^, nisin and EDTA can be used to reduce the uses of sulfite for shrimp preservation in the future. So far, but to the best of our knowledge, the nisin antimicrobial gene has not been transferred to the plant. Nevertheless, some other antimicrobial genes, such as cecropin B, an antibacterial peptide from Bombyx mori, have been transferred to rice to make it more resistant to bacterial leaf blight ^18^. Despite the importance of nisin, the nisin protein has not yet been produced in the plant. Hence, the present study aimed to investigate the production of nisin protein in an indirect regenerated transgenic carrot plant by *Agrobacterium tumefaciens*. Antibacterial activity and preservative properties of the plant extracts were also studied. The carrot plant was chosen because carrots are used directly in some prepared foods. When the nisin gene is express in carrots, it can be directly use as a natural preservative in food, and artificial preservatives are no longer needed. The results can be used as a natural preservative in food products.

## Materials and methods

### Plant material

In vitro plant seedlings were used as an explant source.

### Plasmid construction

The nisin gene sequence of *Lactococcus lactis* was obtained from the NCBI site with code MG913135.1. Codon optimization was performed to express this gene in the plant using the site (http://www.kazusa.or.jp/codon). In order to confirm that the activity of the generated protein did not change after coding optimization, the activity of the optimized protein was also tested by the Blocks site (http://blocks.fhcrc.org)^19^.

**Figure 1:**
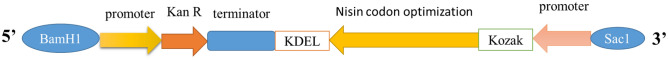
Physical map of recombinant pBI121 plasmid containing the antibacterial nisin optimization gene. Kan R: Kanamycin resistance marker. KDEL: Signal peptide is for protein accumulation in endoplasmic reticulum (ER). Kozak sequence: to increase gene expression in plants. The CaMV 35S promoter. NOS terminator. The nisin gene is from the bacterium *Lactococcus lactis,* which has been optimized for further expression in plants (CDS).

The nisin gene containing the Kozak sequence and the KDEL signaling sequence was first synthesized by the Nedaye Fan company and cloned into the plasmid pBI121 between the Bam*HI* and Sac*I* restriction sites (Fig. 1)^20^. To make the recombinant plasmid, the gene sequence was synthesized with regulatory elements and cutting sites (*Bam*H1/*Sac*1) were given on both sides of the sequence. Then it was cloned in the binary vector pBI121 at the homologous sites. A KDEL signal sequence was added to the end of the gene to collect nisin protein in the endoplasmic reticulum. The nisin gene was under to CaMV35S promoter and NOS terminator. The recombinant pBI121 was first transformed into the *E. coli* strain Dh5α for amplification. Colonies grown in LB medium containing 50 mg/L kanamycin were screened. In the next step, the recombinant plasmid pBI121 was transfected into *Agrobacterium* strain LBA4404 for transformation to the plant^21^. In order to confirm the gene transformation to Dh5α bacteria and *Agrobacterium*, digestion plasmid with *Bam*H1 and PCR were performed.

### Plant material and transformation procedures

After 24–48 h of recombinant *Agrobacterium* growth, 200 μl of the grown bacteria were added to 5 ml of liquid LB medium containing 75 mg/l rifampicin and 50 mg/L of kanamycin and it was placed on a shaker at 28 °C to reach bacterial concentrations (OD) of 0.4, 0.6, 0.8 and 1. Leaf, stem, root, and nodal explants of a 14-day carrot plant were immersed in the bacterial solution for 5, 10, and 15 min. After drying with filter paper, the explants were cultured in the dark for 0. 1, 2, and 3 days in a culture medium (MS salts containing 3% sucrose and 0.8% agar). The explants were then exposed to a callus induction medium containing MS salts, 1 mg/l Naphthaleneacetic acid (optimized for tissue culture) and 50 mg/l kanamycin with a light cycle of 16 h at 28 °C and light intensity was transmitted at 2800 lx. Cefotaxime antibiotics at concentrations of 100, 250 and 500 mg/l were used to remove possible *Agrobacterium* infection.

Five to six weeks after the transfer of explants to callus induction medium, calluses formed in medium containing kanamycin were transferred for embryogenesis in MS medium containing 1 mg/l NAA, 50 mg kanamycin, and cefotaxime for 2–3 weeks. The embryos were transferred to a similar medium to produce seedlings. Seedlings were transferred to ½MS medium for rooting. The light conditions were the same at all stages of growth (temperature was 28 °C, and 16 h of light was 8 h of darkness). To transfer the seedlings to the pot in a ratio of 1: 1: 1 Sand: Vermiculite: Peat was used and every day the seedlings were sprayed with water to form carrot roots and form a complete plant. The percentage of callus induction, embryogenesis and shooting were assessed at all stages.

### Polymerase chain reaction analysis

For the initial screening of transgenic plants and the initial confirmation of the presence of the gene and non-contamination of bacteria, the polymerase chain reaction method with nisin gene-specific primers, kanamycin marker-specific primers, and Vir G gene primers were performed. For this purpose, DNA was first extracted from the leaf of putative transgenic plants using the modified Dellaporta method^4,22^.

The primers sequence used for PCR for NPTII markers, nisin gene and Vir G gene are listed in Table 1.

**Table 1:** Sequence of forward and reverse used primers.

Primer	Forward primer sequence	Reverse primer sequence
NPTII	3′-GAACAAGATGGATTGCACGC-5′	5′-GAAGAACTCGTCAAGAAGGC-3′
NISIN	3′-CTGCTACTTGCCATTGCTC-5′	5′-AGCCTCTCTAACCATCTGTG-3′
VirG	3′-ATGCCCGATCGAGCTCAAGT-5′	5′-TCGTCTGGCTGACTTTCGTCATAA-3′

In order to ensure that putative transgenic plants were not infected with *Agrobacterium*, the leaf DNA extracted by vir gene-specific primers was examined by polymerase chain reaction.

The PCR conditions for each primer are listed in Table 2.

**Table 2:** The cycle program followed these steps.

	*npt*II (Ċ/min)	Nisin (Ċ/min)	*Vir gen* (Ċ/min)
Initial denaturation	94/2 min	94/2 min	94/5 min
Denaturation	94/1 min	94/1 min	94/1 min
Annealing	55/1 min	62/1 min	58/1 min
Extension	72/3 min	72/1 min	72/1 min
Final extension	72/5 min	72/5 min	72/7 min
Cycle	35	35	35

### Dot-blot assay

DNA dot blotting was performed to confirm gene integration in the plant genome using a specific probe of the nisin gene^23^.

### RT-PCR analysis

According to its instructions, the total RNA of leaf carrot plants was extracted by an RNA extraction kit from Pars Toos Company according to its instructions. The extracted RNA of the leaf's extracted RNA was used to synthesize the EURx two-step cDNA synthesis kit. After cDNA preparation, PCR was performed by nisin gene primers.

### Antiserum production in mice

For producing antiserum, the first eight mice were prepared. Four mice were selected as controls, and four were selected for injection. In the first stage, blood samples were taken from all mice before injection.

In order to prepare the protein, 0.01 g of pure nisin protein (Pure nisin was purchased from Sigma) was dissolved in 10 ml of saline phosphate buffer. Aluminum hydroxide was used as an adjuvant. Thirty μl of protein dissolved in saline phosphate was mixed with 60 μl of adjuvant, and 30 μl was injected into each mouse.

Blood samples were taken 1 week after each injection. Four stages of injection and four stages of blood sampling were performed. After each blood sampling, to separate the blood plasma, the vial containing blood was first placed at 37 °C for 20 min, then centrifuged for 10 min at 4000 rpm, and the blood plasma was separated at − 20 °C. Bradford test was performed to confirm antiserum production^24^.

In order to perform the next steps of the work, 12 different dilutions of antiserum were prepared to determine the best dilution of antiserum.

Total protein was extracted from the leaf of putative transgenic carrot plants by Laemmli et al. method^25^. To confirm the expression of the nisin gene. Bradford method concentrated the leaf protein extracted^24^. Then, to confirm the expression of the transferred gene, the Enzyme-Linked Immunosorbent Assay (ELISA) test was performed using by Bradford method^24^. Eight transgenic plants validated by PCR were used for the ELISA test.

### Assay the inhibitory effect of nisin by agar overlay assay

The agar overlay assay method was used to evaluate the antibacterial properties of the nisin protein expressed from the target gene. For this purpose, the first protein was extracted from leaves of transgenic and non-transgenic plants by Laemmli et al. method^25^. SDS PAGE gel was prepared, and the leaf protein extracted was electrophoresis. After running, a gel containing leaf protein extracted from the control and transgenic plants was stained to identify existing bands. Another gel containing leaf protein extracted from control and transgenic plants was prepared to determine the antibacterial properties of nisin protein by the Ko method^26^.

### Protein extraction

In order to evaluate the antimicrobial properties of total protein, it was extracted from leaves from control and transgenic plants by Laemmli et al. method^25^.

### Quantitative assay of sensitivity to serial dilution method

First, the minimum inhibitory concentration (MIC) of leaf protein extracted in the control and transgenic plant was determined, and then the minimum lethal concentration (MBC) was determined. In order to perform the mentioned experiment, concentrations of 500, 250, 125, 62.5, 31.25, 15.63, and 7.81 (µg/ml) were used. The MIC result was evaluated based on the turbidity of the culture medium, which indicates bacterial growth^27^.

### Assay diameter of inhibition for MIC and MBC values

In order to determine the minimum lethal concentration after determination of MIC (concentration of antibiotic that inhibits bacterial growth), falcons containing serial diluted were cultured with swap on LB agar medium. The plates were incubated for 37–18 h at 37 °C, and no bacterial growth was indicated MBC (MBC is the minimum concentration of antibacterial peptide that kills bacteria). The physiological serum was used for negative control and kanamycin for positive control. In order to evaluate the antimicrobial effect of leaf protein extracted from control and transgenic plants, disk diffusion and disc blank methods were used^27^. Kanamycin for positive control and physiological serum for negative control were used in disk diffusion and disc blank forms. A diameter of inhibition more significant greater than 11 mm means no growth, inhibition 8–10 mm and inhibition 5–7 mm is average control and inhibition less than 5 mm is considered non-inhibitory^27^.

### Antimicrobial assay of leaf extracts

For this purpose, first methanolic and aqueous from leaf extracts of the transgenic and non-transgenic plants were extracted^28,29^.

### Assay the stability of leaf protein extracted

For this purpose, two pasteurized orange and peach juices were used. Pasteurized juices were obtained from a local shop in Tehran. Bacteria used included *Escherichia coli* (strain O157:H7) and *Staphylococcus aureus* (strain O157:H7). Concentrations of leaf protein were extracted from transgenic plants containing nisin and non-transgenic plants with the maximum lethality and were used in previous tests obtained in fruit juice (500 μg/ml). After put of juices for 0, 4, 8, 12, 20, 25, 30, 35, 40, 45, 50, 55, 60, 65, and 70 days in the refrigerator (4 °C) and ambient temperature (25 °C), the samples were tested for antibacterial activity. A concentration of 0.5 McFarland from *Escherichia coli* and *Staphylococcus aureus* was spread on an LB agar medium to evaluate the effect of the used protein. A hole approximately 5 mm in diameter was drilled in the center of the petri dish and 25, microliters of treated juices were added to the hole. Then, the Petri dishes were exposed to 37 °C for 24 h, and then the inhibitory zone was examined^30,31^. Nisin-free fruit juice containing bacteria was used as a negative control, and fruit juice without bacteria and nisin was used as a control^32,33^.

### Assay of the effect of leaf protein extracted as preservatives

The bacteria used in this test are *Escherichia coli* and *Staphylococcus aureus*. The concentration of protein at which the maximum bactericidal concentration was obtained was used in this test. For this purpose, the method of Junior et al*.* and Sumonsiri were used^32,33^.

### Analysis of differences in compounds between transgenic and non-transgenic plants

For this purpose, leaf methanolic extract of transgenic and non-transgenic plants was prepared from carrot leaves. Leaf methanolic extract was prepared using by Dhanani method^29^. Then, the measurable compounds were investigated by high-performance liquid chromatography (HPLC) in transgenic and non-transgenic plants.

### Genetic stability assessment

Eighteen ISSR primers were used for DNA replication and analysis^34^ (Table 3). To determine the genetic stability of the transgenic plant compared to the non-transgenic plant. In this experiment, one non-transgenic plant and nine transgenic plants were used. The PCR conditions are given in Table 4.

**Table 3:** ISSR primers used in this experiment.

Primers	Primer sequence
ISSR 1	TTCTTCTTCTTCTTCTTCG
ISSR 3	TCTCTCTCTCTCTCTCC
ISSR 5	TCTCTCTCTCTCTCTCT
ISSR 7	GAAGAAGAAGAAGAAGAAG
ISSR 8	GGAAGAAGAAGAAGAAGAA
ISSR 9	GAAGAAGAAGAAGAAGAAC
ISSR 10	CACACACACACACACAG
ISSR 12	CACACACACACACACAA
ISSR 13	CACACACACACACACAT
ISSR 14	CTCTCTCTCTCTCTCT
ISSR 15	CTCTCTCTCTCTCTCTCA
ISSR 16	CTCTTCTTCTTCTTCTTCT
ISSR 17	TCTTCTTCTTCTTCTTCTC
ISSR 18	TCTTCTTCTTCTTCTTCTG
ISSR 19	CTTCTTCTTCTTCTTCTTC
ISSR 20	CTTCTTCTTCTTCTTCTTG
ISSR 21	CTTCTTCTTCTTCTTCTTC
ISSR 22	CCTTCTTCTTCTTCTTCTT
ISSR 23	GCGAAGAAGAAGAAGAA

**Table 4:** The cycle program followed these steps.

	*npt*II (Ċ/min)
Denaturation	94/1 min
Annealing	53, 55, 59/1 min
Extension	72/1 min
Final extension	72/5 min
Cycle	35

### Statistical analysis

Analysis of Variance (ANOVA) was performed based on a Completely Randomized Design (CRD) with three replications. Data were analyzed using SPSS software. Significantly different means were identified using Tukey’s test (*P* = 0.05).

### Ethics approval and consent to participate

This study did not use wild plants, and carrot seeds (*Daucus carota* L. cv. Nantaise) were prepared from Seed and Plant Improvement Institute (Karaj, Iran). After sterilization of seeds, seeds were cultured in ½MS medium. After 2 weeks, root, shoot, leaf and nodal explants of plantlet were used. This study was conducted at Shahid Beheshti University, faculty of life science and biotechnology, and the biotechnology laboratory, and all processes comply with relevant institutional, national, and international guidelines and legislation. In this study not used of wild plants. This study was conducted at Shahid Beheshti University, Faculty of Biological Sciences and Biotechnology, Biotechnology Laboratory and all of them are in accordance with institutional, national and international guidelines and laws.

## Results

### Plasmid construction

**Figure 2:**
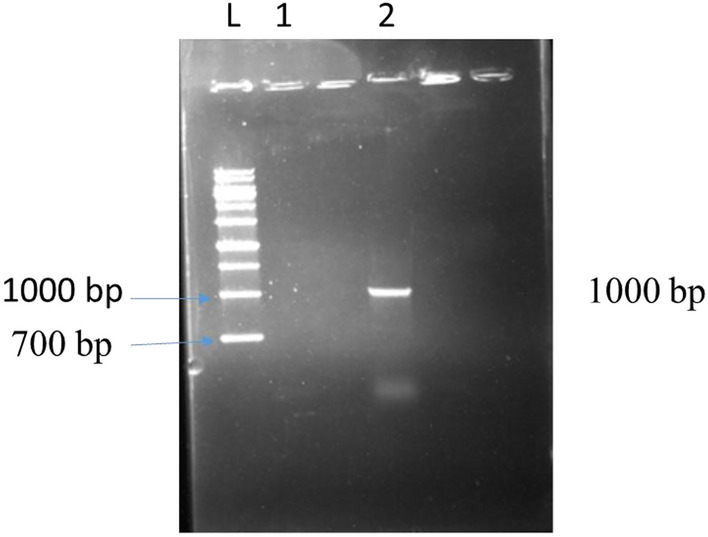
The product of the polymerase chain reaction using specific primers of the nisin gene in a recombinant plasmid. L: Molecular marker. 1: non-recombinant plasmid. 2: The 1000 bp band indicates the presence of the desired gene in the recombinant plasmid.

In order to confirm the recombinant plasmid, the polymerase chain reaction method with specific primers of the nisin gene and enzymatic digestion was used. Specific primers of the nisin gene were detected in band 1000 bp in the PCR product (Fig. 2, Lanes 2). However, in the negative control, no bands were observed, indicating the absence of the desired gene in plasmid (Fig. 2, Lane 1).

**Figure 3:**
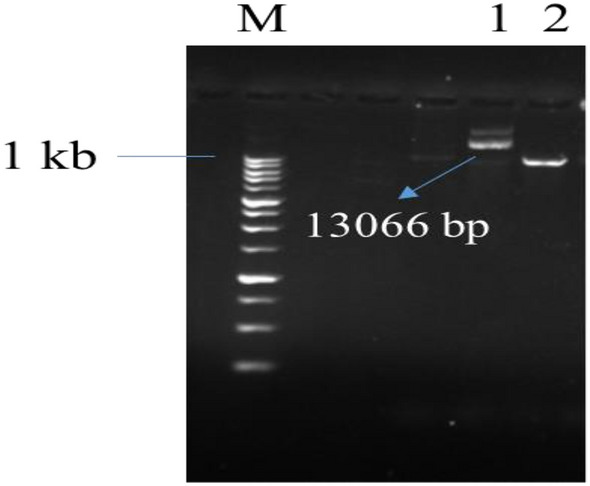
Digest for recombinant plasmid detection. L: 1 Kbp molecular marker. 1: Digested recombinant plasmid. 2: non-digested recombinant plasmid.

In addition, the desired fragment in the recombinant plasmid was detected using enzymatic digestion with band 13,066 bp. Land 2 is the undigested plasmid, which is circular (Fig. 3, Lanes 1 and 2).

### Integration of the nisin gene

**Figure 4:**
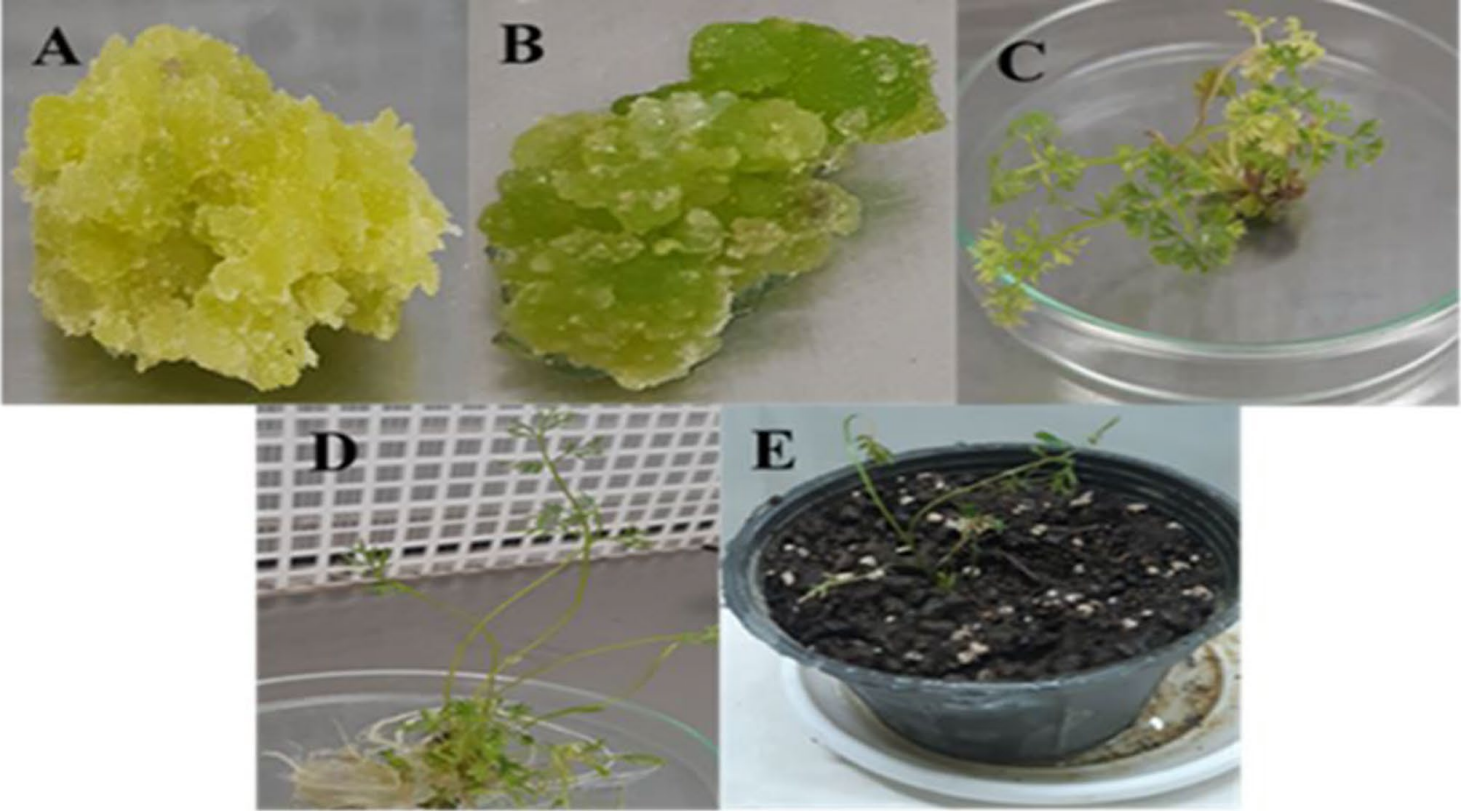
Stages of callus induction and regeneration of carrots in selective medium. (**A**) Induction of transgenic callus induction in the selective medium. (**B**) Transgenic embryogenesis in the selective medium. (**C**) Regeneration of transgenic carrot in the selective medium. (**D**) Rooted Seedling from shoot formed in selectivemedium. (**E**) Transgenic plant in pots.

After immersion of the explants in the inoculum fluid and placing the explants in the selective medium, the explants that survived in the selective medium were cultured every 2 weeks for callus induction, embryogenesis, and shooting products in the same medium as the previous medium (Fig. 4A–E).

**Figure 5:**
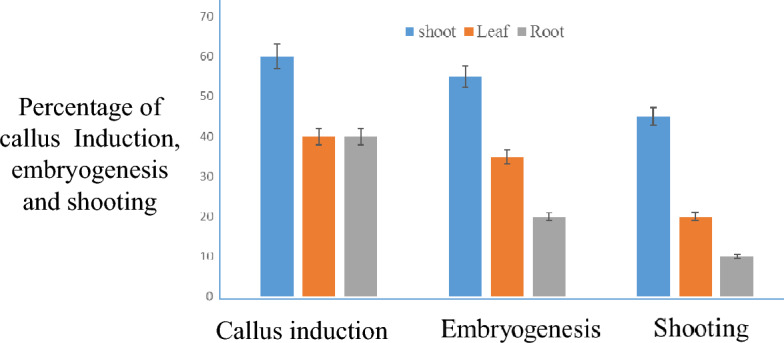
Percentage of callus induction, embryogenesis and shooting from used four explants (shoot, root, leaf and nodal). In selective culture medium.

At these stages, different traits were measured so that the highest percentage of callus induction (60%), embryogenesis (55%), and seedling production (45%) in the selective environment were obtained from stem explants in a concentration of 0.6 to 0.8, 50 mg/l kanamycin and 250 mg/l cefotaxime. The lowest callus induction, embryogenesis and shooting (0%) were obtained from the nodal explants. After shoot explants, root explants and then leaf explants have the highest rates of callus induction, embryogenesis, and shooting, respectively (Fig. 5).

The best result was obtained from inoculum fluid with a 0.6–0.8 *Agrobacterium* concentration in an LB medium containing 50 mg/l kanamycin and 250 mg/l cefotaxime. At a concentration of 0.4%, transgenicity decreased sharply, and at a concentration of 1, the percentage of transgenics decreased due to the contamination of explants (Table 5). PCR obtained the percentage of putative transgenic from the number of plants in which transgenic has been proven concerning the total number of regeneration plants in the selective medium. The best control of *Agrobacterium* without a decrease in the transgenic plant was obtained from 250 mg/l cefotaxime. Contamination decreased the transgenic percentage at a concentration of 100 mg/l cefotaxime due to *Agrobacterium*. Furthermore, at a concentration of 500 mg/l, the inhibitory effect on the growth of explants decreased the transgenic percentage (Table 6).

**Table 5:** Percentage of transgenics^a^ in different concentrations of Agrobacterium (OD).

OD	Percentage of transgenics
0/4	15
0/6–0/8	60
1	35

^a^The number of plants with the desired gene to the total number of plants grown in the selective medium.

**Table 6:** Percentage of transgenic plants in the use of different concentrations of cefotaxime.

Concentration of cefotaxime (mg/l)	Percentage of transgenic (%)
100	20
250	60
500	30

**Figure 6:**
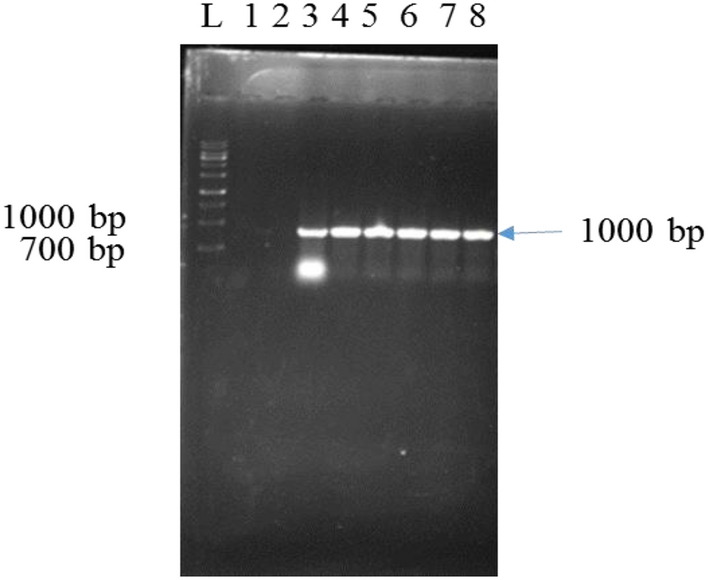
PCR analysis of DNA isolated from leafs of transformed carrot using primer pairs specific for amplification of 1000 bp nisin gene in agarose gel. 1: DNA from untransformed carrot. 2: sterile water. 3: DNA from pBI121-nisin. 4–8: DNA from putative transgenic carrot lines. L: 1.0 kb plus DNA ladder.

**Figure 7:**
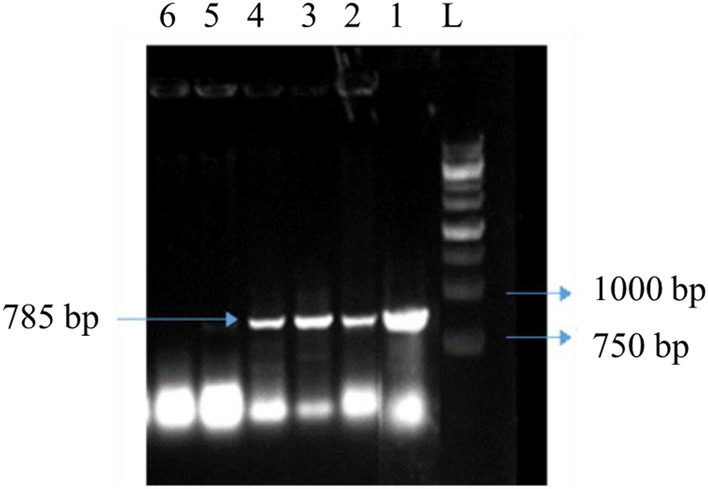
PCR analysis of DNA isolated from leaves of transformed carrot using primer pairs specific for Amplification of 785 bp nptII gene in agarose gel. L: 1.0 kb plus DNA ladder. 1: DNA from pBI121-nisin. 2–4: DNA from putative transgenic carrot lines. 5: DNA from untransformed carrot. 6: sterile water.

This experiment used, three inoculation times, including 5, 10, and 15 min. At the time of inoculation for 15 min, the percentage of transgenics (30%) decreased due to infection of the explants with *Agrobacterium* and tissue loss. However, at 5 and 10 min of inoculation, the transgenic percentage was higher (60%), and no *Agrobacterium* infection was observed. In two inoculation times (5 and 10) were not different in transgenicity.The polymerase chain reaction results from fifty plants grown in the selective medium. Forty-three of fifty plants showed the desired bands amplified by specific nisin gene primers and kanamycin marker primers. In four of the fifty plants, only the amplified band appears with specific primers of the nisin gene, and in three of the fifty plants, only the amplified band appears with specific primers of the kanamycin gene. Neither of these two bands appears in the control plant (Fig. 6 (Lane 4–8), Fig. 7 (Lane 2–4)). The presence of these two bands in transgenic plants compared to the control plant indicates the presence of at least one copy of the nisin gene in transgenic plants.

**Figure 8:**
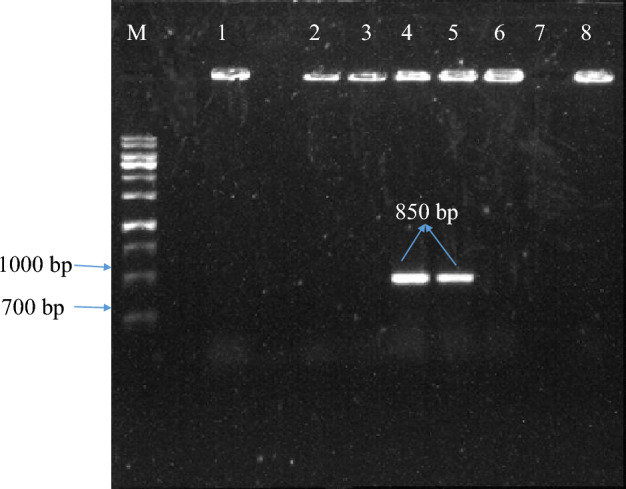
PCR analysis of DNA isolated from leaf of transformed carrot using primer pairs specific for amplification of 850 bp *vir* gene in agarose gel. M. 1.0 kb plus DNA ladder. 1. Sterile water. 4, 5: Agrobacterium containing recombinant pBI121-nisin. 2, 3. DNA from untransformed carrot. 6–8. DNA from putative transgenic carrot lines.

The presence of a 1000 bp band in the polymerase chain reaction was demonstrated using specific primers of the nisin gene. In order to prove the absence of *Agrobacterium* contamination in putative transgenic plants, a polymerase chain reaction was performed using specific virG gene primers. The 850 bp band in the *Agrobacterium* sample and its absence in putative transgenic plants indicates no bacterial contamination (Fig. 8. Lane 4–8). Finally, this study found, that the transgenic percentage was 60%. Then transgenic plants were adapted. The percentage of plant adaptation was 55%.

### Dot blot assay

**Figure 9:**
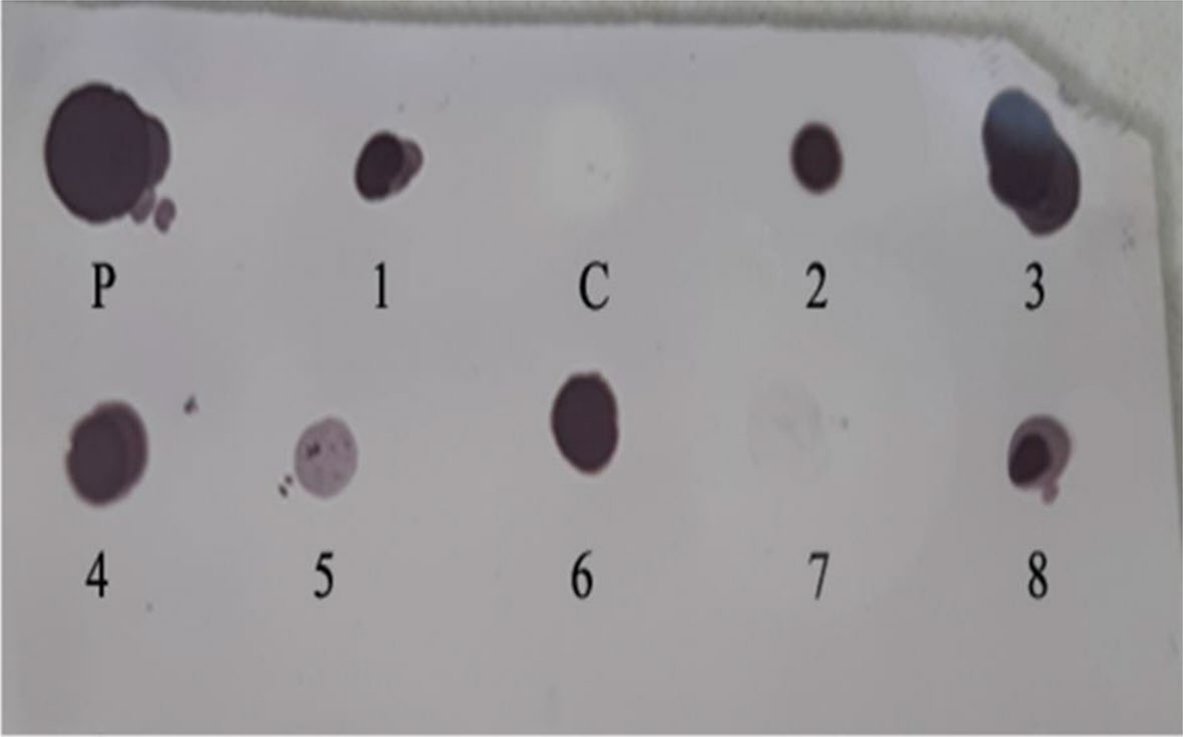
Dot blot analysis of selected transgenic plantlets. P (Plasmid): Positive control from plasmid, C (Control): non-transgenic plantlet, 1–8: transgenic lines.

In a DNA blot test, probe hybridization to genomic DNA confirms the integration of the nisin gene into the carrot genome. However, in the non-transgenic plant, no signal was detected by hybridization. In order to perform DNA dot blotting, lines whose PCR was positive for nisin gene-specific primers were used. In one of the eight lines whose PCR was positive, no signal was detected in the DNA dot blot test, which is probably due to the contamination of this one line with *Agrobacterium,* and this one line was deleted (Fig. 9. Lane: 1–8).

### RT_PCR analysis

**Figure 10:**
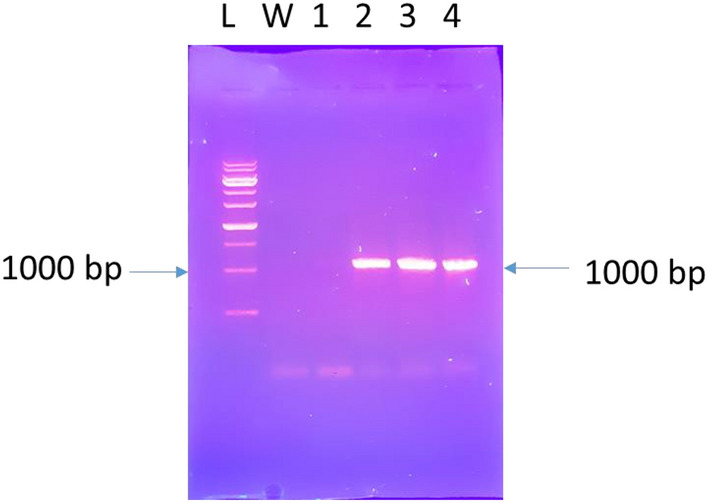
RT-PCR analysis of transgenic plants (nisin: expected size 1000 bp). L: 1.0 kb plus DNA ladder. W: sterile water. 1: untransformed carrot. 2: pBI121-nisin. 3–4: transgenic carrot lines.

RT_PCR analyzed transgenic lines that were tested and confirmed. PCR results of cDNA confirmed the expression of the nisin gene in transgenic lines (Fig. 10 Lane 3–4), but as expected, it was not observed any band in the non-transgenic line as a negative control and water (Fig. 10 Lane 1).

### Antiserum production in mice

Bradford test was performed to confirm antiserum production^24^. The results confirm the production of anti-serum in mice (Table 7). The antiserum concentration produced in mice was 0.74 μg/ml. The best antiserum dilution for subsequent tests was 1: 50 (1.50).

**Table 7:** Antiserum production in mice.

Repetition	No treatment mice μg/ml	Treatment mice (μg/ml)			
		Mice 1	Mice 2	Mice 3	Mice 4
1	0^a^	0.797^b^	0.741^b^	0.625^b^	0.605^b^
2	0^a^	0.881^b^	0.630^b^	0.683^b^	0.685^b^
3	0^a^	0.750^b^	0.667^b^	0.756^b^	0.722^b^

^a^ is in one group and ^b^ is in one group in terms of significance.

### Expression of the nisin gene

ELISA results showed that the nisin gene was the expression in transgenic plants (Table 8). In addition, the amount of nisin protein produced was determined by ELISA. It was founded that in transgenic plants, the amount of nisin expressed in one gram of transgenic plant is from 0.05 to 0.08 μg/ml. Statistical analysis showed that the difference between nisin in control and transgenic plants was significant at 5%.

**Table 8:** Expression of the nisin gene.

Repetition	Non transgenic plants μg/ml	Transgenic plants (μg/ml)				
		Line 1	Line 2	Line 3	Line 4	Line 5
1	0^a^	0.059^b^	0.057^b^	0.065^b^	0.065^b^	0.069^b^
2	0^a^	0.062^b^	0.081^b^	0.068^b^	0.085^b^	0.079^b^
3	0^a^	0.057^b^	0.067^b^	0.075^b^	0.072^b^	0.079^b^

^a^ is in one group and ^b^ is in one group in terms of significance.

### Assay the inhibitory effect of nisin by agar overlay assay

**Figure 11:**
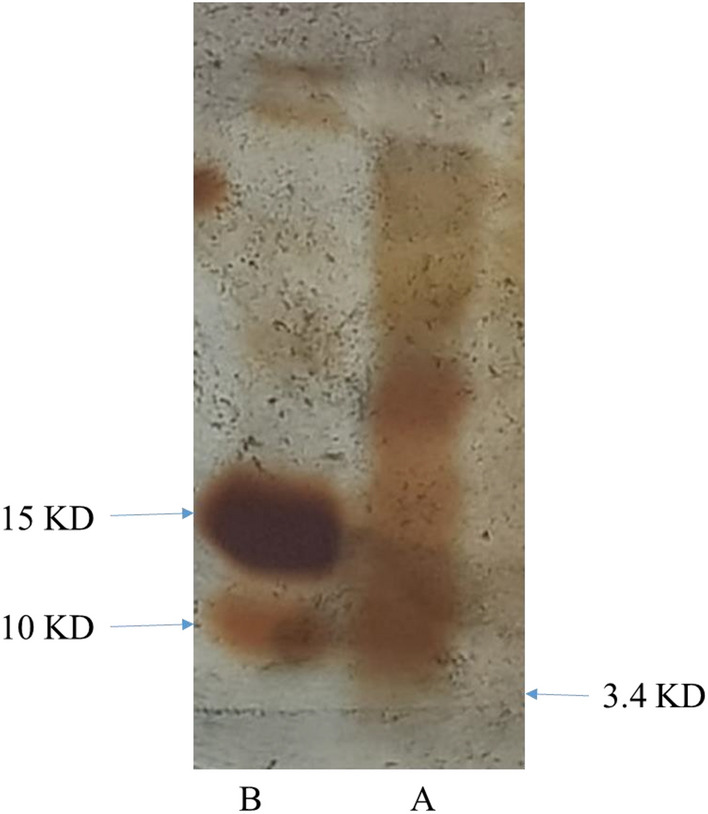
SDS page gel results. (**A**) Transgenic plant containing 3.4 kD band, (**B**) Control plant without 3.4 kD band.

**Figure 12:**
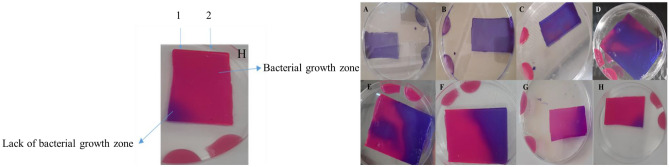
Assay of antibacterial properties by Agar Overlay assay. SDS page gel. 1: Protein extracted from transgenic plant with 3.4 KD band (nisin protein) (blue color indicates antibacterial properties of nisin protein). 2: Leaf Protein extracted from control plant. (**A**–**H**) Steps of SDS page gel staining over time.

The molecular weight of the nisin gene is 3.4 KD. SDS PAGE gel shows a 3.4 KD bands in the transgenic plant, but no 3.4 KD band in the control plant (Fig. 11A, B). The 3.4 KD band indicates the expression of the transferred nisin gene in the transgenic plant. After staining SDS PAGE gel with Resazurin Sodium Salt reagent, the areas where the bacteria have grown are pink and the areas where the bacteria have not grown are blue. The area of the 3.4 KD band, which became pink in the transgenic plant, indicated the expression of nisin protein and the antibacterial properties of nisin protein (Fig. 12A–H).

### The inhibitory effect of nisin on gram-positive and gram-negative bacteria in vitro

**Figure 13:**
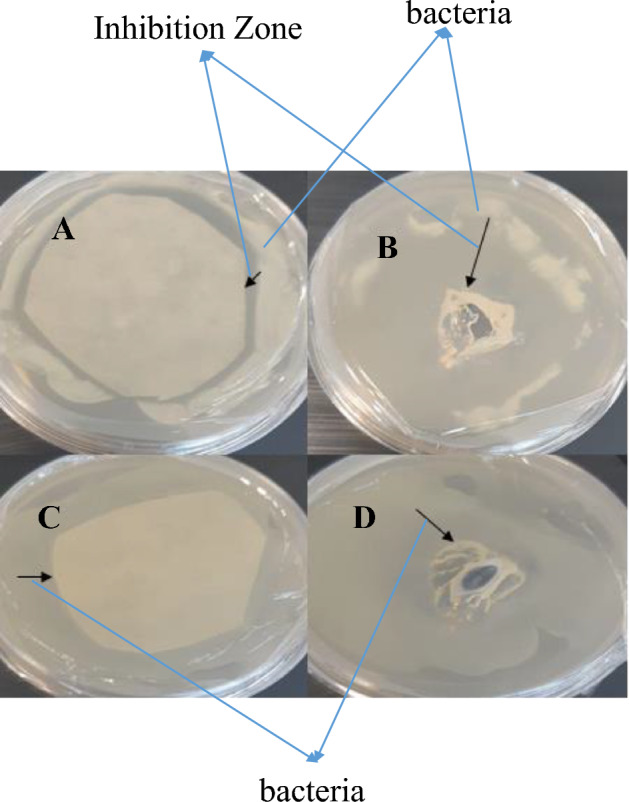
Inhibitory zone in Gram-positive bacteria of *Staphylococcus aureus.* (**A**) Using disc diffusion carrier of leaf protein extracted from transgenic plant, (**B**) Using disc blank containing leaf protein extracted from transgenic plant. (**C**, **D**) Negative control using leaf protein extracted from control plant.

**Figure 14:**
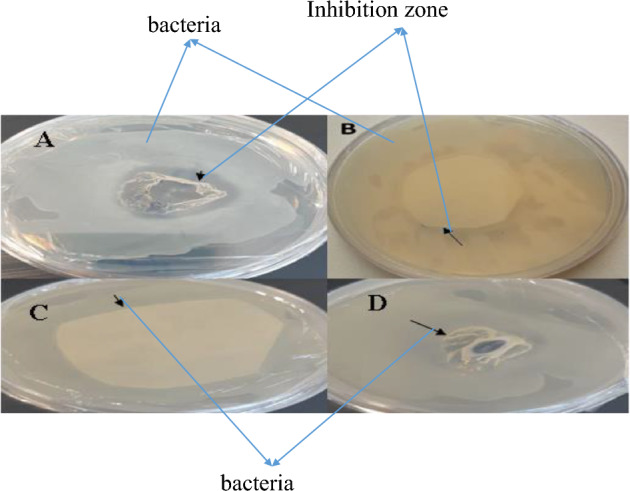
Inhibitory area in *Escherichia coli* (gram-negative bacteria). (**A**) Using disc blank containing leaf protein extracted from transgenic plant. (**B**) Using disk diffusion containing protein derived from transgenic plant. (**C**, **D**) Negative control using leaf protein extracted from control plant.

No inhibition was observed in either gram-positive or gram-negative bacteria of protein extract in control plants (Figs. 13C–D, 14C–D). However, the results of leaf protein extract from the transgenic plant revealed inhibition in gram-positive and gram-negative bacteria (Figs. 13A–B, 14A–B). The results of antimicrobial effects of nisin and comparison with tetracycline antibiotics by disk diffusion in agar and disc blank showed that the diameter of the growth zone created by these compounds in *Staphylococcus aureus* (gram-positive bacteria) is more significant than *Escherichia coli* (gram-negative bacteria). The results showed that nisin significantly affects gram-positive bacteria more than gram-negative bacteria. The diameter of the inhibitory area by nisin was more prominent in both bacteria than in antibiotics.

In *Staphylococcus aureus*, the maximum inhibitory area was 19 mm in the disc blank method and 23 mm in the disc diffusion method. Measurements made with a ruler (Fig. 13A, B). In *Escherichia coli*, the maximum inhibition zone was 9 mm based on the blank disc and blank diffusion methods (Fig. 14A, B).

The results of MBC and MIC experiments showed that the degree of inhibition of bacterial growth by a protein containing nisin was directly related to the amount of protein in the dilutions. By increasing the amount of protein in each dilution, the number of bacterial colonies decreases after culture, and by increasing the amount of protein in each dilution, the number of bacterial colonies decreases after culture. At a concentration of 500 μg/ml leaf protein extract, complete inhibition of *Staphylococcus aureus* was performed (Fig. 13A, B), but this concentration did not completely inhibit *Escherichia coli*, that indicating a lower effect of nisin on gram-negative bacteria (Fig. 14A, B). The minimum inhibitory concentration was obtained in *Staphylococcus aureus* (gram-positive) at 125 μg/ml, but the lowest inhibitory concentration was obtained in *Escherichia coli* at 250 μg/ml.

In addition, in *Staphylococcus aureus,* based on the disc diffusion and disk blank methods, the minimum area inhibition is equal to 5 and 1 mm, respectively. In *Escherichia coli*, the lowest inhibitory areas were obtained in the disc diffusion and disk blank methods of 2 and 1 mm, respectively.

In MIC and MBC determination, none of the leaf aqueous extract dilutions inhibited the growth of *Escherichia coli* and *Staphylococcus aureus* (Table 9).

**Table 9:** Inhibition of leaf aqueous extract.

Bacteria	Inhibition of leaf aqueous extract (mm)
*Staphylococus aureus*	3
*Escherichia coli*	1

However, in this method, leaf methanolic extract in dilution of 1.64 inhibited the growth and death of bacteria, and as MIC and MBC were effective on both *Escherichia coli* and *Staphylococcus aureus* and the two bacteria were not different in terms of MIC and MBC.

**Figure 15:**
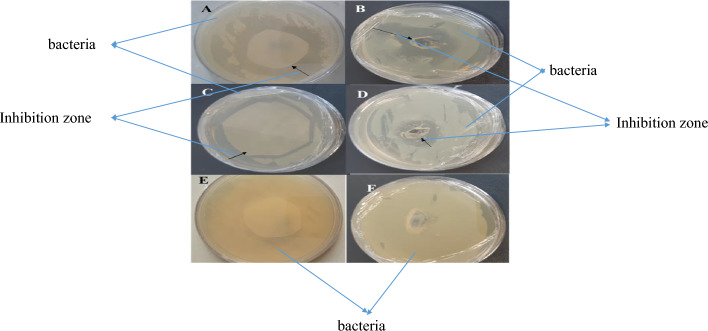
(**A**) Inhibitory region in gram-positive bacteria (*Staphylococcus aureus*) using disk diffusion containing leaf methanolic extract of transgenic plant. (**B**) Inhibition area in *Staphylococcus aureus* using gram-positive bacteria by leaf methanol extracted from the transgenic plant by disc blank. (**C**) Inhibition area in gram-negative *Escherichia coli* using disc diffusion containing leaf methanolic extract, extracted from the transgenic plant. (**D**) Area inhibition in *Escherichia coli* gram-negative bacteria using disc blank containing leaf methanolic extract, extracted from transgenic plant. (**E**, **F**) Negative control using leaf methanolic extract, extracted from control plant. Black arrows indicate inhibitions.

The highest inhibitory area of *Staphylococcus aureus* was obtained by leaf methanolic extract using the 19 mm disk diffusion and 16 mm disc blank methods. In addition, in *Escherichia coli*, the highest inhibitory area was obtained by leaf methanolic extract by the 15 mm disk diffusion method and by the 11 mm disk blank method (Fig. 15A–F).

### Assay the stability of leaf protein extracted

The antibacterial activity of leaf protein extract of transgenic carrot against two bacteria, *Staphylococcus aureus* and *Escherichia coli,* in peach and orange juices up to 70 days after treatment of juices and storage at two temperatures of 37 °C and 4 °C was checked (Table 10). When the shelf life of the treat juices was extended to 70 days, no change in the inhibitory value of leaf protein extract of transgenic was observed in either orange or peach juices against both bacteria at 4 °C, and no significant difference was observed between these treatments at this temperature. However, at 25 °C on day 70, no change was observed in the inhibitory amount of leaf protein extract of transgenic in orange juice against both bacteria. Nevertheless, the inhibitory amount of nisin on peach juice at 25 °C in the 70th day was reduced against both bacteria, but this decrease was more noticeable against *Escherichia coli*. The difference between these treatments was significant at a 5% level. No inhibition was observed in control juices in which nisin was not used.

**Table 10:** Stability assessment of leaf protein extract of transgenic carrot.

Bacteria	Days		0		4		8		12		20		25		30		35		40		45		50		55		60		65		70	
*Staphylococus aureus*	Temperature °C		4	25	4	25	4	25	4	25	4	25	4	25	4	25	4	25	4	25	4	25	4	25	4	25	4	25	4	25	4	25
	Inhibition of area (mm)	Orange juice	ns 1 ± 20	ns 20 ± 1	ns 20 ± 1	ns 20 ± 1	ns 20 ± 1	ns 20 ± 1	ns 20 ± 1	ns 20 ± 1	ns 20 ± 1	ns 20 ± 1	ns 20 ± 1	ns 20 ± 1	ns 20 ± 1	ns 20 ± 1	ns 20 ± 1	ns 20 ± 1	ns 20 ± 1	ns 20 ± 1	ns 20 ± 1	ns 20 ± 1	ns 20 ± 1	ns 20 ± 1	ns 20 ± 1	ns 20 ± 1	ns 20 ± 1	ns 20 ± 1	ns 20 ± 1	ns 20 ± 1	ns 20 ± 1	ns 20 ± 1
		Peach juice	ns 18 ± 2	ns 18 ± 2	ns 18 ± 2	ns 18 ± 2	ns 18 ± 2	ns 18 ± 2	ns 18 ± 2	ns 18 ± 2	ns 18 ± 2	ns 18 ± 2	ns 18 ± 2	ns 18 ± 2	ns 18 ± 2	ns 18 ± 2	ns 18 ± 2	ns 18 ± 2	ns 18 ± 2	ns 18 ± 2	ns 18 ± 2	ns 18 ± 2	ns 18 ± 2	ns 18 ± 2	ns 18 ± 2	ns 18 ± 2	ns 18 ± 2	ns 18 ± 2	ns 18 ± 2	ns 18 ± 2	ns 18 ± 2	**9 ± 2
*Escherichia coli*	Temperature °C		4	25	4	25	4	25	4	25	4	25	4	25	4	25	4	25	4	25	4	25	4	25	4	25	4	25	4	25	4	25
	Inhibition of area (mm)	Orange juice	ns 10 ± 2	ns 10 ± 2	ns 10 ± 2	ns 10 ± 2	ns 10 ± 2	ns 10 ± 2	ns 10 ± 2	ns 10 ± 2	ns 10 ± 2	ns 10 ± 2	ns 10 ± 2	ns 10 ± 2	ns 10 ± 2	ns 10 ± 2	ns 10 ± 2	ns 10 ± 2	ns 10 ± 2	ns 10 ± 2	ns 10 ± 2	ns 10 ± 2	ns 10 ± 2	ns 10 ± 2	ns 10 ± 2	ns 10 ± 2	ns 10 ± 2	ns 10 ± 2	ns 10 ± 2	ns 10 ± 2	ns 10 ± 2	ns 10 ± 2
		Peach juice	ns 1 ± 8	ns 8 ± 1	ns 8 ± 1	ns 8 ± 1	ns 8 ± 1	ns 8 ± 1	ns 8 ± 1	ns 8 ± 1	ns 8 ± 1	ns 8 ± 1	ns 8 ± 1	ns 8 ± 1	ns 8 ± 1	ns 8 ± 1	ns 8 ± 1	ns 8 ± 1	ns 8 ± 1	ns 8 ± 1	ns 8 ± 1	ns 8 ± 1	ns 8 ± 1	ns 8 ± 1	ns 8 ± 1	ns 8 ± 1	ns 8 ± 1	ns 8 ± 1	ns 8 ± 1	ns 8 ± 1	ns 8 ± 1	** 6 ± 2

### Assay of the effect of leaf protein extracted as preservatives

**Figure 16:**
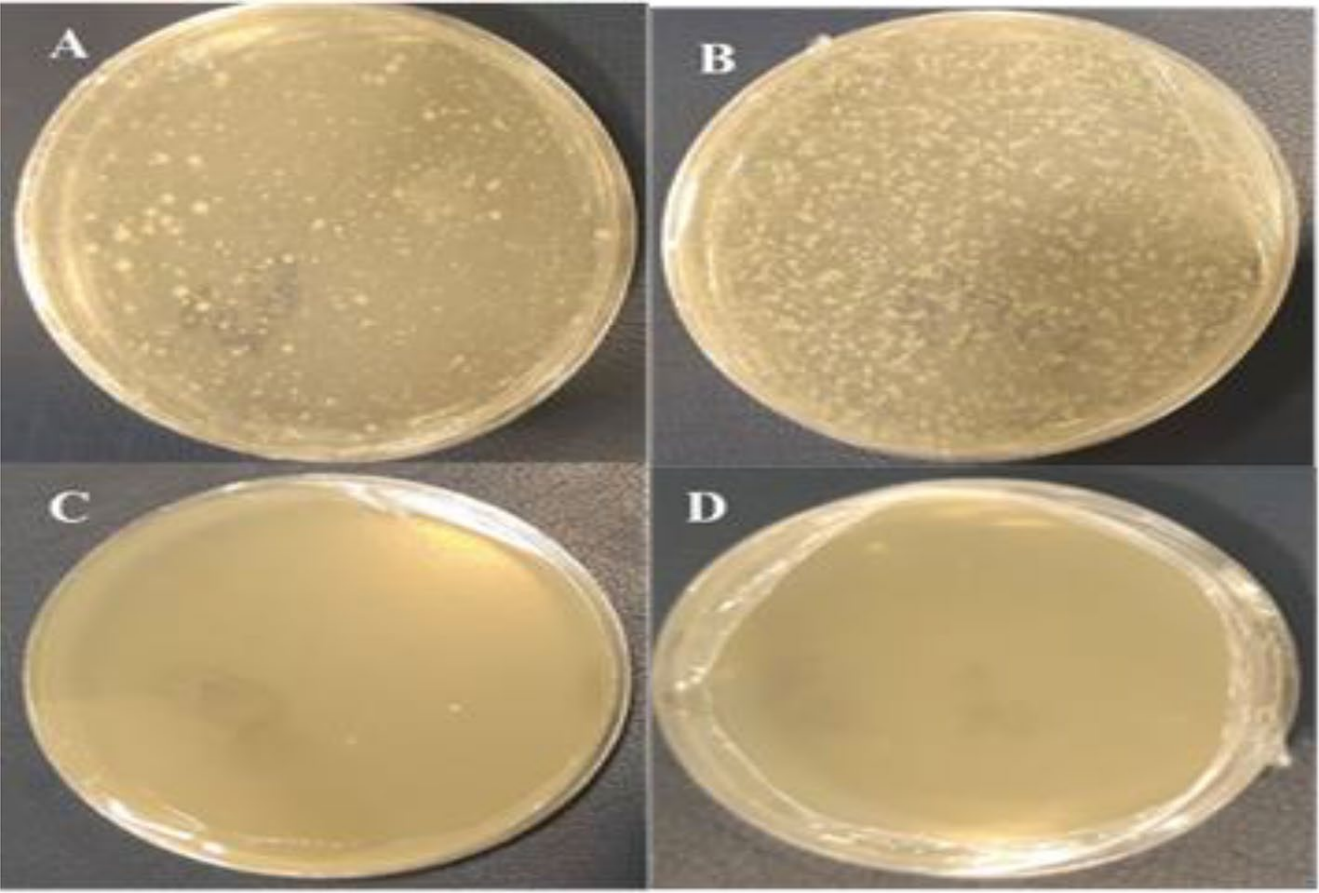
Treatment of orange juice with *Escherichia coli* and *Staphylococcus aureus* with a concentration of 106 CFU/ ml and leaf protein of transgenic plant. (**A**) Treatment of orange juice with *Escherichia coli* at 0 h, (**B**) Treatment of orange juice with *staphylococcus aureus* at 0 h, (**C**) Orange juice treatment with *Escherichia coli* at 24 h, (**D**) Orange juice treatment with *staphylococcus aureus* at 24 h.

In orange juice at zero time, 106 CFU/ml were obtained using *Staphylococcus aureus* and *Escherichia coli*. At a time of 8 h, 2 log was obtained in the use of *Staphylococcus aureus* and three log in the use of *Escherichia coli*. Complete inhibition of both bacteria *Staphylococcus aureus* and *Escherichia coli* was also achieved after 24 h of use (Fig. 16A–D).

**Figure 17:**
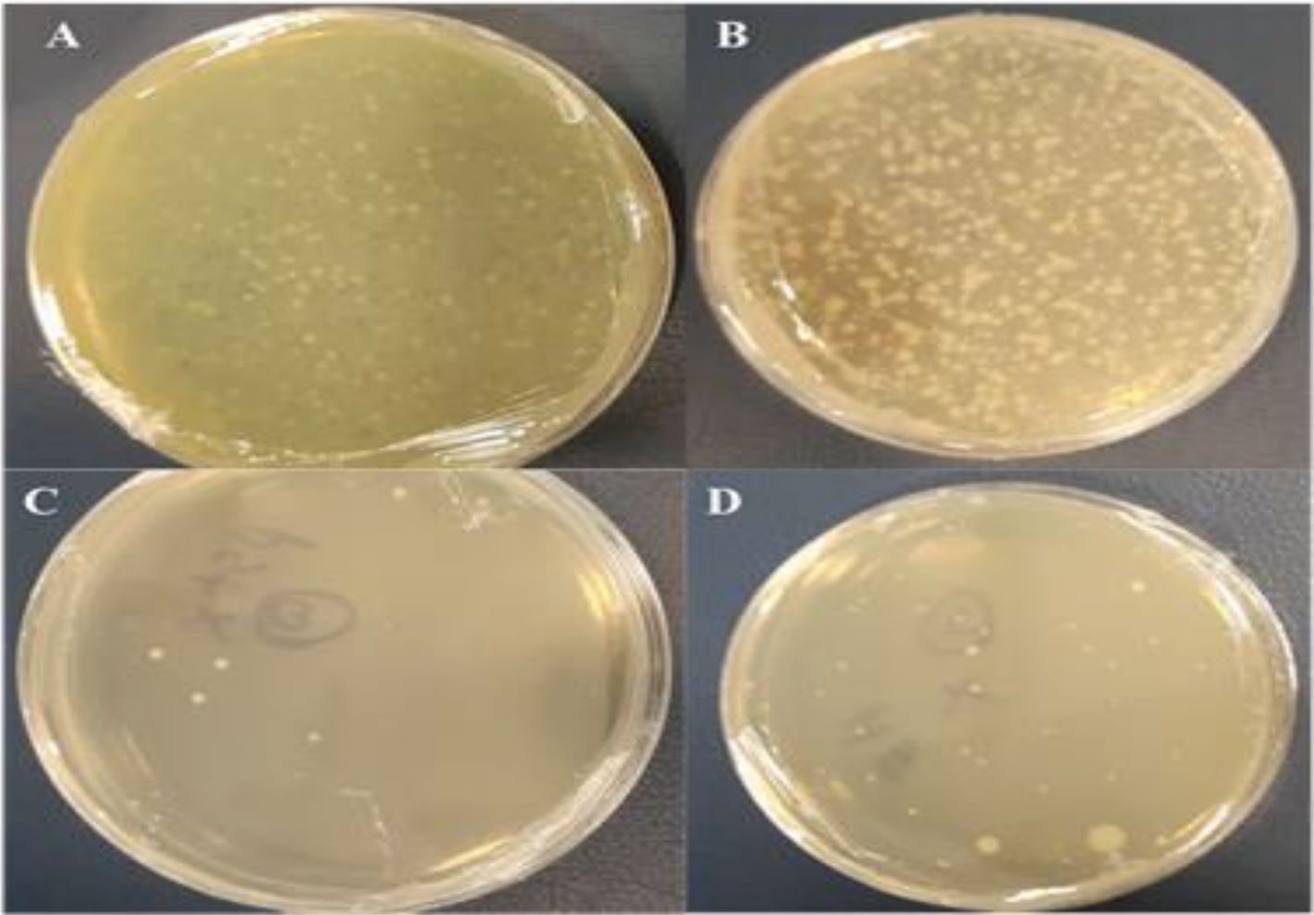
Peach juice treatment with *Escherichia coli* and *Staphylococcus aureus* with 106 CFU/ml concentration and leaf protein extract of transgenic plants containing nisin gene. (**A**) Peach juice treatment with *Escherichia coli* at 0 h, (**B**) Peach juice treatment with *Staphylococcus aureus* at 0 h, (**C**) Peach juice treatment with *Escherichia coli* at 24 h, (**D**) Peach juice treatment with *Staphylococcus aureus* at 24 h.

Peach juice was obtained at 106 CFU/ml at zero time using *Staphylococcus aureus* and *Escherichia coli*. At 8 h, logs 2 of *Staphylococcus aureus* and logs 3 of *Escherichia coli* were obtained. After 24 h, log 1 was obtained in peach juice containing *Staphylococcus aureus* and logs 2 in peach juice containing *Escherichia coli* (Fig. 17A–D).

### Analysis of differences in compounds between transgenic and non-transgenic plants

In this study, 18 compounds were analyzed. Results of HPLC indicate, Chlorogenic and Chicoric acid compounds were increased in transgenic plants, but this increase was not significant. However, 16 other combinations (Galic, 3.4dhb, catchin, valnic acid, caffiec, 2.5 dhb, sirgnic, coumaric, ferulic acid, rutin, rosmarinic acid, salicylic acid, quercetin, cinnamic acid, kaempferol and apigenin) did not show a difference between transgenic and non-transgenic plants.

### Genetic stability of regeneration carrot plants

**Figure 18:**
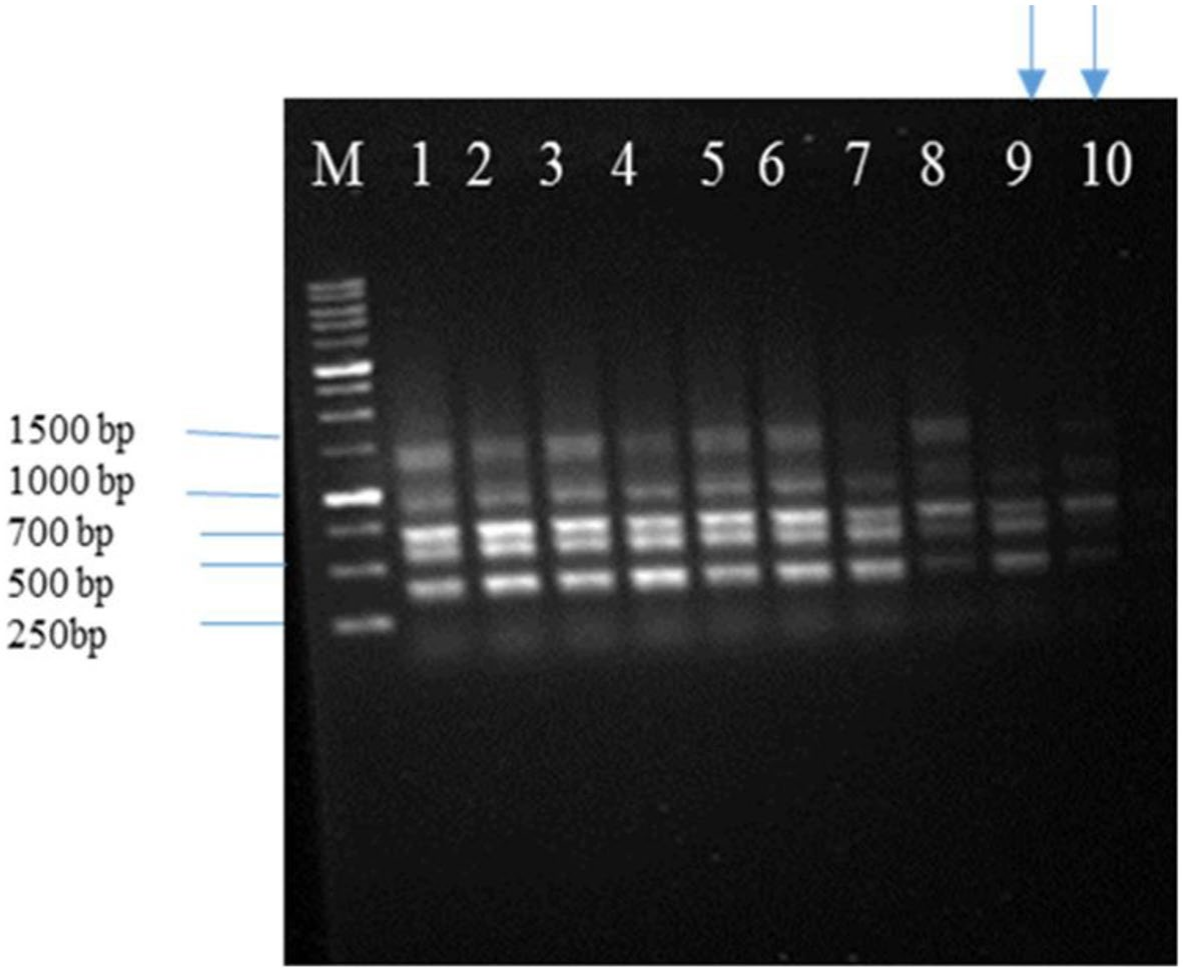
Evaluation of genetic stability of carrots using ISSR marker. Profiles were obtain with ISSR 10 primer in carrot plant. M. Marker, 1. Non-transgenic plant, 2–10. Transgenic plants.

Nineteen ISSR primers were used in this experiment to evaluate genetic stability. All bands formed by ISSR primers used were the same between transgenic plants and non-transgenic plants except for plants 9 and 10 of transgenic plants (Fig. 18, lanes 1–10).

## Discussion

Transgenic carrot plants using the transformation system described previously^35,36^ were readily obtained in this investigation.

At first, for optimization gene transformation to carrot plants, it is necessary to determine the optimal concentration of inoculum containing *Agrobacterium* and the concentration of cefotaxime and kanamycin antibiotics. At less than optimal concentrations, non-transgenic plants may also grow, at over-optimized concentrations, transgenic plants' regeneration may be prevented. As a result, optimization of antibiotic concentration is needed to increase the efficiency of gene transformation^37^.

In the study, Tokuji et al. used ½MS liquid medium containing an *Agrobacterium* plate with OD 0.8 as inoculum^35^. However, the study by Yadav et al., used a liquid LB medium containing *Agrobacterium* with OD 0.7^38^. The results of both studies were similar to the results of the present study.

This study’s results are similar to those of Yadav et al.^38^ for the dilution of *Agrobacterium* and concentration of kanamycine and cefotaxime. In contrast Baranski et al. used 100 mg/L kanamycin. In the study of Pandey et al., the best cell density for gene transformation in cumin plant was 0.6^39^. Seemingly, this amount of dilution achieves the most active stage of logarithmic growth and multiplication of *Agrobacterium* strains^38^. Among other things, the type of *Agrobacterium* strain has been used to increase the efficiency of gene transfer. The present study used, *Agrobacterium tumefaciens* strain LBA4404 was used and the most promising best results were obtained from stem explants. Similarly, Hardegger and Sturm^36^ used the same *Agrobacterium* strain in their research. Nevertheless, Roderick et al.^40^ used the C58C1 strain^41^ and Yoshihiko et al.^41^ used the EHA101 strain to transform the gene into the carrot. In many experiments, the LBA4404 strain has been identified as the most efficient strain^38–42^. Different strains of *Agrobacterium* have different abilities to transformation genes to different plant species and genotypes within a species^43^.

Another critical factor in the effectiveness of gene transformation is the duration of inoculation of explants in *Agrobacterium* solution. Accordingly, it depends on the type and age of the explant, plant species and even the plant genotype used, *Agrobacterium* strain, type and concentration of culture medium compounds, method of inoculation and inoculation temperature^43^. Research usually ranged 2–60 min for inoculation time.

In addition, the times used for inoculation in this study are similar to the results of Haji Heidar et al., in 2015^44^. However, Tokuji et al. in 1999 inoculated the explants for 2 h^35,41^.

Moreover, the results of co-culture time obtained from this study are similar to the results obtained from the research of Mohjel Kazemi et al. and Hardegger et al.^36^. Tokuji et al. used 5 days of, co-cultivation, and the percentage of transgenicity was obtained at 60%^41^. In Baranski's study, gene transfer efficiency was 90%, the transformation rate was 3.8^45^, in the study of Ghaboli et al., 62%^40^, in the study of Roderick et al., and 10% in the study of Tokuji et al.^46^. Reasons for different results include differences in the type of *Agrobacterium* used, the type of variety used, and the use of different explants^37^.

In previous reports, RT_PCR has been used to evaluate mRNA expression^47–49^. In this research, RT_PCR was used to confirm the expression of the nisin gene in the transgenic plant. In RT_PCR, was found that the nisin gene was expressed in the studied lines. PCR, RT_PCR, and DNA dot blot results confirmed that the nisin gene was integrated into the genome. Correspondingly, to further confirm gene expression, an ELISA test was performed. ELISA test was performed and the results revealed that the integrated nisin gene in the genome of transgenic lines had the ability of transcriptional activity, and the resulting mRNA was translated. ELISA is a suitable test for identifying specific proteins in living organisms. It has been the most widely used serological method for detecting and identifying of plant pathogens such as bacteria and viruses and has been used to detect and measure the number of proteins expressed in transgenic plants.

In this experiment, the Agar Overlay assay was used to confirm the antibacterial properties of nisin. This study, confirmed the antibacterial properties of nisin protein against gram-positive bacteria *Staphylococcus aureus* by Agar Overlay assay technique.

Nuthan et al. used the Agar Overlay TLC—Bioautography Assay technique to detect metabolites for pharmaceutical applications^50^. Furthermore, Hockett et al. used the Soft-agar Overlay technique to observe bacterial inhibitory compounds^51^.

Moreover, the leaf protein extracted from transgenic and non-transgenic plants was used to investigate the antibacterial properties of nisin protein. The leaf protein extracted from the control plant did not show any inhibition, either in both gram-positive and negative bacteria. However, leaf proteins extracted from transgenic plants showed inhibition in both gram-positive and negative bacteria. The effect of leaf protein extracted from the transgenic plant containing nisin on the gram-positive bacterium *Staphylococcus aureus* was greater and significant than the gram-negative bacterium *Escherichia coli*. Leaf protein extract of transgenic carrot is effective on bacterial membranes, and because gram-negative bacteria have two membranes, the effect of nisin peptide on it is limited^5,52^.

This study, as in previous studies, showed that with increasing the concentration of the extract, the antimicrobial activity of the extract increases^53^. According to Mostafa et al.^54^, the difference in MIC of plant extracts is due to their diversity of chemical compounds and volatiles. Likewise, aqueous and methanolic extracts of transgenic and non-transgenic plants were used to investigate the antibacterial properties of nisin protein. Previous findings have shown that alcoholic extracts are more antimicrobial than aqueous^54^. Al-Hashimi et al.'s^54^ results are similar to those obtained in this study. In addition, it has been reported that a large number of different chemical compounds (such as phenolic compounds and their derivatives, esters, fatty acids, terpenes, and other compounds) are present in the leaf methanolic extract of the spice, and therefore, these chemical compounds can affect several target sites^55,56^. Similar results were observed for MIC values with slight changes in other dimensions^54,57,58^.

After confirming the antibacterial property of the nisin protein expressed in the plant, the stability of the protein extracted from the transgenic plant containing nisin as a preservative and its antibacterial property was tested in peach and orange juice. So far, a limited number of studies have examined the effect of nisin on fruit juices^59^. The results of Walker et al. and Pathanibul et al. show the effect of nisin in apple juice on the control of *Listeria innocua* and *A. acidoterrestris*^60,61^. Adelson et al. proved that nisin could kill *S. aureus, A. acidoterrestris, L. monocytogenes and B. Inhibit cereus* strains in fruit juice^32^. Furthermore, in research by Lorenzo et al., nisin powder was used to prevent the growth of *Listeria monocytogenes* in herbal drinks^16^. The use of bacteriocins as preservatives in fruit juices depends on factors such as peptide solubility, bacteriocin interactions with fruit juices, juice pH, and inactivation by proteases^62,63^. Therefore, the stability of bacteriocin in fruit juice is crucial. This study found that nisin can control bacteria up to a concentration of 106 CFU/ml.

These results indicated that the transgenic carrot leaf protein extract could remain stable in juices, control bacteria, and makes the juices microbiologically safe.

In addition, it was found that nisin's antimicrobial activity is not the same and depends on the type of juice and the microorganisms present in the juice^32^.

The results of previous research showed that the effect of nisin on *A. acidoterrestris* (gram-positive bacteria) is greater than on *L. monocytogenes* (gram-positive)^64–66^. Another study found, that nisin had a greater effect on *Staphylococcus aureus* (gram-positive) than *Escherichia coli* (gram-negative)^67^. Because nisin can cause pores on the cytoplasmic membrane of bacteria, especially gram-positive bacteria^68^. The formation of these pores in the cytoplasmic membrane can prevent the proton from moving and prevent the pH balance, which in turn leads to ion leakage, ATP hydrolysis, and, ultimately bacterial cell death. In addition, nisin can bind to lipid II, a precursor to peptide and glycan synthesis, and inhibit cell wall biosynthesis^33^. Accordingly, these results are similar to the results of the present study.

Pei et al. and Yamazaki et al. report that the antibacterial activity of bacteriocins in different juices is not the same, due to the composition of bacteriocins and polyphenols in the juice^63,69^.

Fruits such as peaches, mangoes and almonds have been shown to contain divalent ions such as calcium and magnesium^70^. These dual-capacity ions have been shown to reduce bactericidal activity^71–73^. In addition, it was reported that binding divalent ions maintain nisin stability to ribitol and glycerol phosphate. Therefore, the different activity of nisin depends on the juice and the type of microorganisms^71^. These results revealed the reasons for the difference in nisin stability in peach and orange juices in the control of *Staphylococcus aureus* and *Escherichia coli* in the present study, and it was consistent with the results of the present study.

The concentration of nisin should be such that in addition to controlling microorganisms, it does not change the taste and color of the juice that this condition was obtained with the concentration used in this experiment.

In the next step, the genetic stability of the transgenic plants was investigated. Production of transgenic plants and somaclonal diversity resulting from plant regeneration can lead to the diversity of metabolic compounds in transgenic and non-transgenic plants^74^. Metabolic variability between transgenic and non-transgenic plants may be due to the location of the transferred gene^74^. Sometimes these unwanted changes can have beneficial effects^74^.

Modir Rosta et al.'s research found a significant difference between some compounds (oily compounds, glucose content, two amino acids) of the BT transgenic plant and the non-transgenic cotton plant. However, no significant difference was between sucrose and 18 amino acids. In the present study, the two compounds differed from those in transgenic and non-transgenic plants. One of the reasons for the difference between these compounds could be the location of the gene integration. Other reasons for this difference of compounds, related to the regeneration process, could be causes of metabolic variations between the transgenic and non-transgenic counterparts^75^. Further research is needed for this purpose.

The result of genetic stability using ISSR primers showed high genetic stability between the mother plant and the plant regenerated by somatic embryogenesis in the carrot plant. Previous studies have reported dependent that genome-dependent diversity, the location of the organ in the seedling, organs, the age of the organ on the seedling, and the type and concentration of plant growth regulators^76–78^.

In this study, the cause of diversity in regenerated plants maybe somaclonal diversity in somatic embryogenesis in tissue culture^78,79^. The reason for somaclonal diversity is related to the laboratory tissue culture system. Cell division and cell wall formation in somatic embryogenesis are greater than in direct organogenesis. Because of this, somaclonal diversity in somatic embryogenesis is greater than in direct embryogenesis^76–82^.

The present study demonstrated that nisin could be used as a natural preservative in food. Besides, this study showed that recombinant nisin significantly affects controlling pathogenic bacteria, especially gram-positive bacteria. Due to the non-allergenic study peptides, they can be used in research and after the research as a natural preservative in food and medicine. Previously, pure nisin was used as a preservative. In this study, for the first time, recombinant nisin with codon optimization is produced in carrot plants with strong regulatory elements and it was found that it has significant effects in controlling bacteria. These plants can be directly used in food, and there is no need to use preservatives in food. The reason for using carrots is that the production of nisin in the fruit itself, such as apples or oranges, is expensive and time-consuming because the tissue culture of woody plants such as apples takes a long time. In the case of carrot, its growth period is short, while its production rate is also high, because the biomass of carrot, which is the root, is high. The reason for not using the bacteria itself is that, first, the production in bacteria is low, secondly, its purification is costly, while in carrots, the production in the root is high. Second, it does not need to be purified because the carrot root is powdered and consumed directly in food.

All the transgenic carrots produced in this research are containment. According to Iran's Biosafety law any GMO that is Containment does not require a permission. Only GMO that are going to be commercialized or released need a permission. Currently the transgenic carrots produced in this research as a GMO are containment in greenhouse.

## Data Availability

All data generated or analyzed during this study are included in this published article (and its supplementary information files).
